# Combined therapy strategies of immune checkpoint inhibitors in gynecologic oncology: progress and challenges

**DOI:** 10.3389/fonc.2026.1860901

**Published:** 2026-07-16

**Authors:** Xiaomei Hu, Mingshu Zhou, Weimin Kong

**Affiliations:** 1 Department of Obstetrics and Gynecology, Capital Medical University Electric Power Teaching Hospital, Beijing, China; 2 Department of Obstetrics and Gynecology, Beijing Obstetrics and Gynecology Hospital, Capital Medical University, Beijing, China

**Keywords:** biomarkers, combination therapy, gynecologic cancers, immune checkpoint inhibitors, tumor microenvironment

## Abstract

Gynecologic malignancies, including cervical, endometrial, and ovarian cancers, remain major threats to women’s health [1]. Despite advances in conventional treatments, outcomes for advanced or recurrent disease remain unsatisfactory [2]. Immune checkpoint inhibitors (ICIs) offer new hope but are limited by low response rates as monotherapy [3]. This review summarizes the application of ICIs in gynecologic cancers, focusing on combination strategies with chemotherapy, radiotherapy, PARP inhibitors, and anti-angiogenic agents, along with their mechanisms and clinical progress [4]. Key clinical trial data are presented to demonstrate the efficacy of combination approaches. Current challenges are also analyzed, including drug resistance, adverse events, treatment optimization, and biomarker development [5]. ICI-based combination therapy has revolutionized the management of advanced gynecologic cancers. The key issue now is how to optimize these regimens in clinical practice, which requires deeper understanding of the tumor immune microenvironment, reliable multi-dimensional biomarkers, and well-designed clinical trials [6].

## 1 Introduction

Gynecologic cancers are among the leading causes of cancer-related mortality in women globally and represent a persistent public health issue. Recent data show rising incidence of endometrial cancer in many developed countries (1). Ovarian cancer is often diagnosed at an advanced stage due to subtle early symptoms and lack of effective screening, resulting in persistently high mortality among gynecologic cancers (2). Cervical cancer incidence has declined significantly in developed countries due to high screening coverage, yet it remains a leading cause of cancer death in regions with limited healthcare resources (1). For patients with advanced or recurrent disease, conventional treatments including surgery, platinum-based chemotherapy, and radiotherapy often yield unsustained responses and poor median survival, especially in platinum-resistant ovarian cancer and metastatic cervical cancer, creating an urgent need for novel therapeutic approaches (2).

**Figure 1:**
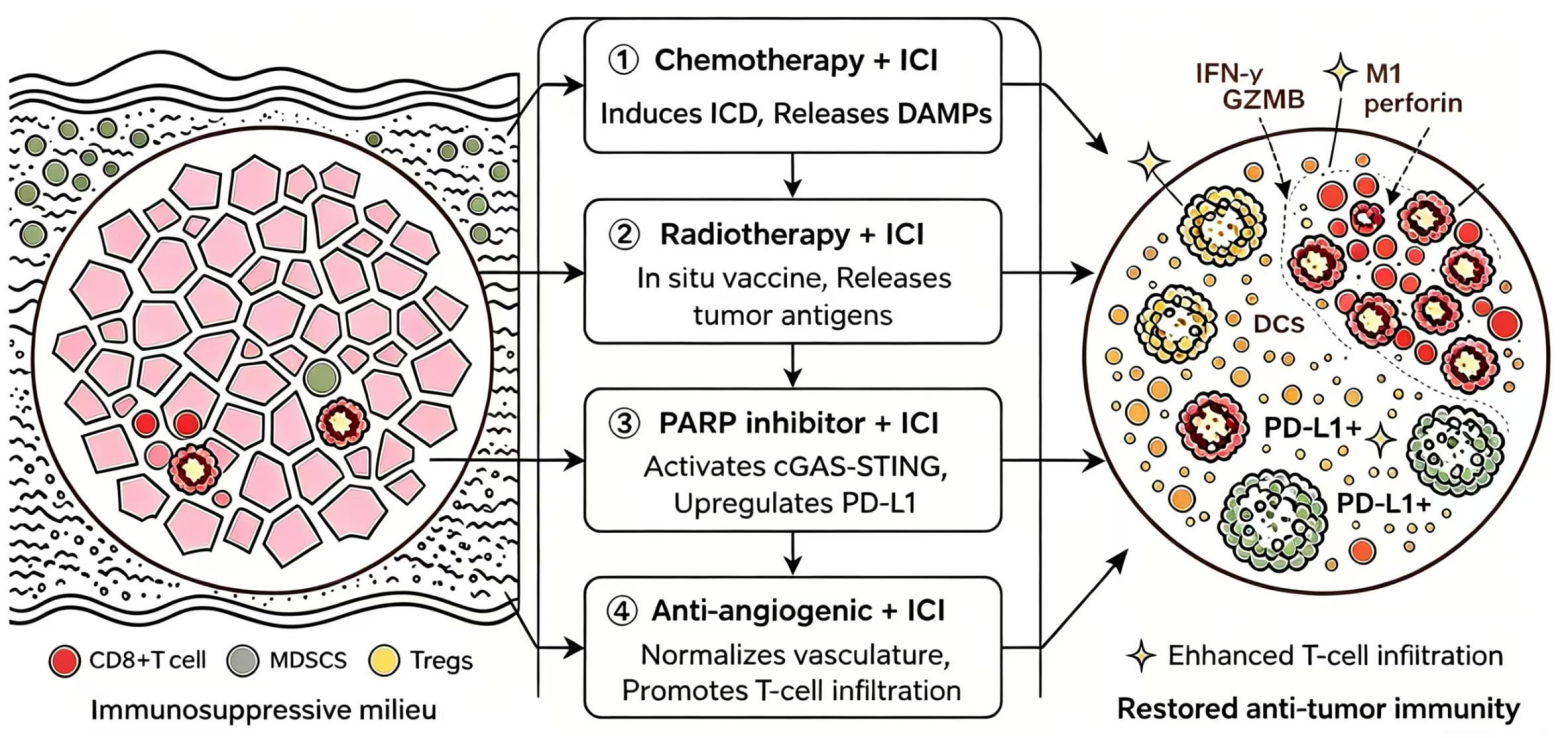
Schematic illustration of “cold-to-hot” tumor conversion by ICI combination strategies. The left panel depicts a “cold” tumor characterized by poor CD8^+^ T-cell infiltration, abundant immunosuppressive cells (TAMs, MDSCs, Tregs), and an immunosuppressive milieu. The middle panel illustrates four synergistic combination strategies that remodel the tumor immune microenvironment (TIME): chemotherapy (induces immunogenic cell death, releases DAMPs), radiotherapy (“*in situ* vaccine”, releases tumor antigens), PARP inhibitors (activates cGAS-STING, upregulates PD-L1), and anti-angiogenic agents (normalizes vasculature, promotes T-cell infiltration). The right panel shows the resulting “hot” tumor with enhanced CD8^+^ T-cell infiltration, DC activation, IFN-γ/GZMB/perforin production, PD-L1 upregulation, and restored anti-tumor immunity.

In recent years, agents targeting immune checkpoints such as PD-1/PD-L1 and CTLA-4 have transformed the landscape of cancer comprehensive therapy (3). ICIs have become standard care for many advanced solid tumors by blocking tumor-induced immune evasion signals and reactivating T cell-mediated anti-tumor immunity, offering new hope and prolonged survival for some patients with advanced gynecologic cancers (3). Nevertheless, with the exception of the MSI-H/dMMR molecular subtype in endometrial cancer, response rates to ICI monotherapy remain low across most gynecologic cancers (7). The primary underlying cause is the high heterogeneity and complexity of the tumor immune microenvironment (TIME), characterized by immunosuppressive cells, inhibitory cytokines, and dysfunctional vasculature (5). Accordingly, research focus has shifted toward combination regimens to overcome the limitations of single-agent ICIs (4). The fundamental approach is to combine ICIs with therapeutics of distinct mechanisms to synergistically remodel the TIME: chemotherapy induces immunogenic cell death, radiotherapy releases tumor antigens, PARP inhibitors activate innate immune pathways, and anti-angiogenic drugs enhance T-cell infiltration into tumors (4). These strategies aim not only to boost ICI efficacy but also to expand clinical benefits to more patients. This review summarizes the mechanisms and clinical advances of ICI-based combination therapies in gynecologic cancers and addresses major mechanistic and translational challenges, with the goal of informing the development of more precise and personalized immunotherapy (**Figure 1**) (6).

## 2 Current status of ICI monotherapy in major gynecologic cancers

### 2.1 Endometrial cancer

Endometrial cancer has the most robust evidence supporting ICI benefit among gynecologic malignancies, with efficacy closely tied to molecular subtype (8). The multicenter, open-label, multi-cohort phase II basket trial KEYNOTE-158 confirmed that single-agent pembrolizumab achieved an objective response rate (ORR) of 57.1% in previously treated patients with advanced MSI-H/dMMR endometrial cancer, with median duration of response (DoR) not yet reached, indicating durable and favorable anti-tumor activity (9). Based on these results, the US FDA approved pembrolizumab for this population, marking the first ICI approved for endometrial cancer (9).

However, approximately 70%–80% of endometrial cancers are microsatellite stable (MSS) or proficient mismatch repair (pMMR), and response rates to ICI monotherapy in this subgroup are generally low, typically below 10%–15% (8). Loss of PTEN function and activating PIK3CA mutations are common in endometrial cancer and may drive an immunosuppressive microenvironment through persistent activation of the PI3K/AKT/mTOR pathway, for example, increasing regulatory T-cell (Treg) infiltration and promoting secretion of inhibitory cytokines such as IL-10 and TGF-β, leading to primary resistance to ICIs (10). In addition, low tumor mutational burden (TMB) and impaired antigen presentation such as downregulation of MHC class I expression are also recognized as important factors affecting treatment efficacy (11).

### 2.2 Ovarian cancer

Ovarian cancer has the worst prognosis among gynecologic cancers (12). Most patients present at an advanced stage; although initially sensitive to platinum-based chemotherapy, up to 70% eventually relapse and develop platinum resistance, with limited subsequent treatment options (2). The TIME of ovarian cancer is often described as “immune-excluded” or “immune-desert,” characterized by scarce infiltration of CD8+ cytotoxic T lymphocytes, abundant accumulation of immunosuppressive cells including Tregs and myeloid-derived suppressor cells (MDSCs), and elevated PD-L1 expression on tumor cells and tumor-associated macrophages (TAMs), collectively creating a highly immunosuppressive microenvironment (13).

Early phase I/II clinical trials evaluating single-agent ICIs including nivolumab, pembrolizumab, and avelumab in recurrent ovarian cancer reported an overall ORR of only approximately 10%–15% and median progression-free survival (PFS) of just 2–3 months, indicating modest efficacy (14). Notably, subgroup analyses revealed that patients who remained platinum-sensitive (platinum-free interval >6 months) derived greater benefit from ICI therapy than platinum-resistant patients (14). This suggests that platinum chemotherapy may “prime” the TIME by inducing immunogenic cell death (ICD), including exposure of calreticulin (CRT) and release of damage-associated molecular patterns (DAMPs) such as high-mobility group box 1 (HMGB1) and ATP (15). This process may prepare the tumor for subsequent immune attack and provides a mechanistic rationale for combining chemotherapy with immunotherapy (15).

### 2.3 Cervical cancer

Cervical cancer has a distinct etiology among gynecologic cancers: nearly all cases are associated with persistent high-risk human papillomavirus (HPV) infection (16). After viral integration into the host genome, sustained expression of the E6 and E7 oncoproteins drives carcinogenesis and serves as ideal tumor-specific antigens for immunotherapy (17). Theoretically, viral antigens should elicit robust immune responses. In practice, however, HPV-associated cervical cancer develops multiple sophisticated immune evasion mechanisms, including upregulation of PD-L1 on tumor and antigen-presenting cells, recruitment of Tregs and MDSCs, and secretion of immunosuppressive cytokines such as TGF-β and IL-10, thereby establishing an inhibitory TIME that allows tumors to escape immune attack (16).

Based on KEYNOTE-158, the FDA approved pembrolizumab for patients with recurrent or metastatic cervical cancer with disease progression during or after chemotherapy and whose tumors express PD-L1 (combined positive score [CPS] ≥1) (18). Nevertheless, in this approved indication, single-agent pembrolizumab achieved an ORR of only 14.3% and median overall survival (OS) of just 9–11 months, indicating substantial room for improvement (18). Combination therapy is now widely regarded as key to improving outcomes of cervical cancer immunotherapy, with major focus on regimens combining ICIs with chemotherapy, radiotherapy, anti-angiogenic agents such as bevacizumab, and other immunomodulators. The core goal is to reverse immune tolerance and convert immunologically “cold” tumors to “hot” tumors (19).

## 3 Mechanisms and clinical advances of ICI combination therapy strategies

### 3.1 ICIs plus chemotherapy

Chemotherapy was traditionally considered immunosuppressive, but recent studies show that certain cytotoxic agents including platinum compounds, taxanes, and anthracyclines exert immune-stimulatory effects under specific doses and schedules (20). Beyond direct tumor cell killing, these drugs can induce a specialized form of cell death known as immunogenic cell death (ICD), which forms the basis for synergy between chemotherapy and immunotherapy (15). The hallmarks of ICD include surface exposure of CRT (“eat-me” signal) and release of DAMPs such as HMGB1 and ATP before cell death. CRT and DAMPs are recognized by dendritic cells (DCs), promoting their maturation and migration to lymph nodes, where they efficiently present tumor antigens to naïve T cells and initiate potent and specific anti-tumor immune responses (15). Importantly, chemotherapy can selectively deplete immunosuppressive cells such as Tregs and MDSCs and upregulate MHC class I molecules and tumor-associated antigens on cancer cells, thereby remodeling the TIME to favor subsequent ICI activity (20). Collectively, these mechanisms enhance tumor immunogenicity and provide a strong rationale for combining ICIs with chemotherapy (**Figure 1**) (15, 20).

#### 3.1.1 Endometrial cancer

Multiple phase III trials have provided pivotal evidence in the treatment of advanced endometrial cancer. The KEYNOTE-775 trial demonstrated that second-line lenvatinib plus pembrolizumab improved patient survival compared with conventional chemotherapy (21). The combination arm had median PFS of 7.2 months versus 3.8 months in the control arm (*HR* = 0.56, *p* < 0.001), reducing the risk of disease progression by 44%; median OS was extended from 11.4 months to 18.3 months, with a 38% reduction in the risk of death (*HR* = 0.62, *p* < 0.001) (21). These data solidified the regimen as a standard of care and confirmed for the first time that targeted-immunotherapy combinations are superior to chemotherapy in MSS/pMMR endometrial cancer, challenging the prior view that this subtype is unresponsive to ICI monotherapy (21).

For first-line treatment, the KEYNOTE-B96 trial evaluating pembrolizumab plus chemotherapy showed significant PFS improvement (*HR* = 0.62, *p* < 0.001) (22). However, another phase III trial (LEAP-001) testing lenvatinib plus pembrolizumab in the first-line setting failed to meet the primary endpoints of PFS and OS compared with chemotherapy. These results underscore the importance of patient selection and biomarker-guided personalized therapeutic strategies (23).

#### 3.1.2 Cervical cancer

The KEYNOTE-826 study represents a milestone in the first-line treatment of persistent, recurrent, or metastatic cervical cancer (18). The study showed that adding pembrolizumab to platinum-based chemotherapy with or without bevacizumab provided clinically meaningful survival benefits. This advantage was particularly pronounced in PD-L1-positive (CPS ≥1) patients: median PFS was 10.4 months in the pembrolizumab arm versus 8.2 months in the control arm (*HR* = 0.62, *p* < 0.001); median OS was significantly prolonged to 24.4 months versus 16.5 months (*HR* = 0.62, *p* < 0.001). This study established pembrolizumab plus chemotherapy (± bevacizumab) as a new first-line standard of care for PD-L1-positive advanced cervical cancer (18).

Beyond first-line metastatic therapy, the KEYNOTE-A18 trial showed that adding pembrolizumab to concurrent chemoradiotherapy significantly improved PFS in patients with locally advanced cervical cancer, expanding the application of immunotherapy to earlier-stage patients (24). In addition, the BEATcc trial confirmed that adding atezolizumab to bevacizumab plus platinum-based chemotherapy yielded dual PFS and OS benefits in patients with metastatic cervical cancer, further consolidating the role of ICI combination therapy in cervical cancer (25).

#### 3.1.3 Ovarian cancer

Exploring ICI-chemotherapy combinations in the first-line treatment of ovarian cancer has proven more challenging than in cervical and endometrial cancers. For example, the phase III JAVELIN Ovarian 100 trial, which evaluated avelumab combined with chemotherapy followed by maintenance therapy, failed to improve PFS and did not meet its primary endpoint (26). This does not mean combination strategies are ineffective in ovarian cancer. Several phase II studies such as TOPACIO/KEYNOTE-162 suggest that ICIs combined with PARP inhibitors or chemotherapy retain efficacy in biomarker-selected populations such as patients with homologous recombination deficiency (HRD)-positive or BRCA-mutant tumors, warranting further investigation (27).

Taken together, clinical evidence indicates inconsistent efficacy of ICI-chemotherapy combinations across gynecologic cancers (18, 21–27). In cervical and endometrial cancers, some combinations have become standard first- or second-line treatments and markedly changed clinical practice (18, 21–25). In first-line ovarian cancer, however, similar approaches have not replicated this success (26). This suggests that intrinsic features of the TIME may fundamentally determine responsiveness to combination therapy (13). It also highlights that future research should focus not merely on testing new combinations, but on deeply understanding the interactions between chemotherapy and immunity across different tumors and molecular subtypes, moving from a “one-size-fits-all” model toward more precise personalized combination therapy (27).

#### 3.1.4 Critical analysis of trial failures: why did promising combinations fail?

The failure of trials such as JAVELIN Ovarian 100 and LEAP-001, despite solid preclinical rationales, offers important lessons for future study design. In ovarian cancer, the lack of a chemotherapy backbone that reliably induces immunogenic cell death (ICD), combined with a poorly infiltrated (“cold”) immune microenvironment characterized by sparse CD8+ T cell infiltration, may explain the disappointing results of first-line ICI combinations (26). In LEAP-001, the failure of lenvatinib plus pembrolizumab to improve outcomes over chemotherapy in unselected endometrial cancer patients underscores the urgent need for biomarker-driven patient selection (23). These observations suggest that future trials should prioritize molecular enrichment strategies and the development of more potent immunogenic chemotherapy regimens to turn “cold” tumors “hot” (27).

### 3.2 ICIs plus PARP inhibitors

PARP inhibitors (PARPis) induce synthetic lethality in tumor cells with defective homologous recombination repair (HRR), such as those harboring BRCA mutations, representing a major breakthrough in ovarian cancer therapy (28). Beyond direct tumor killing, growing evidence reveals prominent immunomodulatory properties of PARPis: DNA damage stress induced by PARPis activates the intracellular cGAS-STING pathway, leading to robust production of type I interferon (IFN-I). IFN-I is a key activator of the immune system, enhancing DC function and promoting recruitment and activation of CD8+ T cells (28). Furthermore, PARPis modify tumor immunogenicity through multiple mechanisms, including increasing TMB and neoantigen load and upregulating PD-L1 expression on tumor cells. Together, these mechanisms provide a strong rationale for combining PARPis with ICIs to achieve synergistic anti-tumor effects greater than the sum of individual agents (28).

While activation of the cGAS-STING pathway is a key mechanism by which PARP inhibitors enhance anti-tumor immunity, additional mechanisms also contribute (28). These include the upregulation of PD-L1 on tumor cells (which paradoxically could limit the efficacy of PD-1 blockade), induction of replication stress and genomic instability, and remodeling of the chromatin landscape to increase antigen presentation. More recently, chronic PARP inhibitor exposure has been shown to drive innate immune adaptation—a state in which tumor cells become less responsive to STING activation, potentially limiting the long-term benefit of PARP inhibitor-ICI combinations (28). This may explain why trials such as the combination of niraparib and dostarlimab in MSS endometrial cancer showed only modest activity (29). Future studies should explore intermittent dosing schedules or combinatorial strategies targeting alternative innate immune pathways (28).

#### 3.2.1 Endometrial cancer

A multicenter phase II trial evaluating niraparib plus the anti-PD-1 antibody dostarlimab in patients with previously treated advanced endometrial cancer showed encouraging results (29). The regimen achieved an ORR of 72% in the dMMR/MSI-H subgroup, demonstrating strong synergy. Remarkably, an ORR of approximately 20%–30% was also observed in the MSS/pMMR subgroup, which is typically unresponsive to immunotherapy. This opens a new therapeutic avenue for these hard-to-treat patients and suggests that immune tolerance can be overcome by inducing DNA damage and activating innate immunity even in the absence of high mutational burden (29).

#### 3.2.2 Ovarian cancer

The phase III DUO-O trial investigated a novel combination maintenance strategy in newly diagnosed advanced high-grade serous ovarian cancer (30). After initial induction with chemotherapy plus durvalumab, patients received maintenance therapy with durvalumab plus olaparib. Results showed that this combination maintenance strategy significantly prolonged median PFS compared with traditional post-chemotherapy placebo maintenance in the HRD-positive subgroup, with a hazard ratio (*HR* = 0.49, *p* < 0.001) (30). This finding strongly supports the potential of PARP inhibitor-immunotherapy combinations to reshape treatment paradigms in precisely selected molecular subtypes (30).

### 3.3 ICIs plus radiotherapy

Radiotherapy is a cornerstone of gynecologic cancer treatment, especially for cervical and locally advanced endometrial cancers. Traditionally viewed as a local modality, mounting evidence now shows that radiotherapy can also trigger systemic immune responses known as the “abscopal effect”—regression of non-irradiated distant metastases following irradiation of one tumor site (31). Three main mechanisms underlie this effect: first, radiotherapy induces ICD of tumor cells, releasing tumor antigens and effectively acting as an “*in situ* vaccine”; second, it upregulates MHC class I molecules and various tumor antigens on cancer cells to enhance immunogenicity; third, it modifies tumor vasculature and the surrounding cytokine milieu to facilitate T-cell infiltration (31).

However, radiotherapy is a double-edged sword: while activating immunity, it can simultaneously induce upregulation of immunosuppressive molecules such as PD-L1 and TGF-β (31). ICIs can precisely block these negative feedback loops, thereby amplifying the immune-stimulatory effects of radiation. Numerous preclinical studies have confirmed synergistic anti-tumor activity of radiotherapy plus ICIs. For example, in mouse tumor models, local radiotherapy plus anti-CTLA-4 antibody controlled not only the irradiated primary tumor but also effectively suppressed growth of distant non-irradiated lesions—an effect dependent on CD8+ T cells (31). In locally advanced cervical cancer, the global phase III CALLA trial evaluated adding durvalumab during and after concurrent chemoradiotherapy (32). Although the primary endpoint (PFS) was not statistically significant in the initial analysis, prespecified subgroup analyses suggested a trend toward PFS benefit in patients with high PD-L1 expression. Multiple phase II studies are ongoing to explore optimal integration of ICIs with chemoradiotherapy in this patient population (32).

### 3.4 Other promising combination strategies

#### 3.4.1 ICIs plus anti-angiogenic agents

Vascular endothelial growth factor (VEGF) is not only a potent pro-angiogenic factor but also a key immunosuppressive mediator. It inhibits DC maturation, promotes expansion and accumulation of Tregs and MDSCs, and impairs effector T-cell function. Anti-angiogenic agents such as bevacizumab and lenvatinib can “normalize” disorganized tumor vasculature, improving perfusion and drug delivery, reducing hypoxia, and alleviating immunosuppression, creating a more favorable microenvironment for T-cell infiltration and function (19). The success of KEYNOTE-775 and KEYNOTE-826 can be partly attributed to this synergy (18, 21). Other agents, including bispecific antibodies targeting VEGF and other pathways, are under active investigation (19).

#### 3.4.2 Dual immunotherapy

Simultaneous targeting of two complementary immune checkpoints, such as PD-1 and CTLA-4, represents another important strategy (33). CTLA-4 inhibitors act primarily in lymph nodes during the early phase of T-cell activation, whereas PD-1/PD-L1 inhibitors function mainly in peripheral tissues and the tumor microenvironment during the effector phase of T-cell activity. The phase II NRG-GY003 study compared ipilimumab (anti-CTLA-4) plus nivolumab (anti-PD-1) with nivolumab monotherapy in recurrent ovarian cancer. The combination arm achieved an ORR of 31.4%, significantly higher than 12.2% in the monotherapy arm, although the rate of grade 3–4 treatment-related adverse events was also increased (33). In addition, combinations of PD-1 inhibitors with agents targeting novel targets such as LAG-3, TIGIT, and TIM-3 are currently in early clinical development (34). The key phase II/III trials of ICIs in gynecologic cancers are summarized in **Table 1**.

**Table 1:** Summary of key phase II/III trials of ICIs in gynecologic cancers.

Trial name	Cancer type	Experimental arm	Control arm	Phase	Biomarker threshold	ORR	Median PFS	Median OS	HR (PFS/OS)	Key findings
KEYNOTE-158	Endometrial (MSI-H/dMMR)	Pembrolizumab	—	II	MSI-H/ dMMR	57.1%	—	—	—	Single-agent pembrolizumab yields durable anti-tumor activity (9).
KEYNOTE-775	Endometrial	Lenvatinib + Pembrolizumab	Chemotherapy	III	MSS/ pMMR	—	7.2 vs 3.8 mo	18.3 vs 11.4 mo	HR=0.56 (PFS), HR = 0.62 (OS)	Combination significantly improves PFS and OS vs chemotherapy (21).
KEYNOTE-B96	Endometrial	Pembrolizumab + Chemotherapy	Chemotherapy	III	All-comers	—	13.1 vs 8.7 mo	—	HR=0.62 (PFS)	First-line chemo-immunotherapy prolongs PFS (22).
LEAP-001	Endometrial	Lenvatinib + Pembrolizumab	Chemotherapy	III	All-comers	—	—	—	—	Failed to meet primary survival endpoints (23).
KEYNOTE-826	Cervical	Pembrolizumab + Chemo ± Bevacizumab	Chemo ± Bevacizumab	III	CPS ≥1	—	10.4 vs 8.2 mo	24.4 vs 16.5 mo	HR=0.62 (PFS), HR = 0.61 (OS)	Standard first-line regimen for PD-L1-positive advanced cervical cancer (18).
KEYNOTE-A18	Cervical	Pembrolizumab + Concurrent CRT	Concurrent CRT	III	All-comers	—	—	—	HR=0.73 (PFS)	Adjuvant pembrolizumab improves PFS in locally advanced disease (24).
BEATcc	Cervical	Atezolizumab + Bevacizumab + Chemo	Bevacizumab + Chemo	III	All-comers	—	—	—	—	Dual PFS/OS benefit with atezolizumab addition (25).
JAVELIN Ovarian 100	Ovarian	Avelumab + Chemotherapy	Chemotherapy	III	All-comers	—	—	—	—	Failed primary PFS endpoint in unselected ovarian patients (26).
DUO-O	Ovarian	Durvalumab + Olaparib Maintenance	Placebo Maintenance	III	HRD-positive	—	—	—	HR=0.49 (PFS)	Maintenance immunotherapy + PARPi improves PFS in HRD+ patients (30).

## 4 Advances in predictive biomarker research

Identification of reliable predictive biomarkers is critical for personalized immunotherapy to maximize clinical benefit and minimize unnecessary toxicity. Research in this field has advanced far beyond reliance on PD-L1 expression alone (35).

### 4.1 PD-L1 expression

Detection of PD-L1 protein expression by immunohistochemistry (IHC), using scoring systems such as combined positive score (CPS) and tumor proportion score (TPS), remains the most widely used clinical biomarker, particularly in cervical and gastric cancers (36). However, its predictive value in gynecologic cancers is inconsistent, and technical challenges related to testing platforms, antibodies, and scoring criteria limit its universal applicability (36).

### 4.2 MSI-H/dMMR status

This hypermutated phenotype caused by DNA mismatch repair deficiency is one of the most effective predictive biomarkers for ICI response. It accounts for approximately 20%–30% of endometrial cancers but is rare in ovarian (<2%) and cervical cancers. Testing for dMMR has become a standard component of endometrial cancer diagnosis (7).

### 4.3 Tumor mutational burden

TMB refers to the number of somatic non-synonymous mutations per megabase of DNA and reflects neoantigen abundance. High TMB (commonly defined as ≥10 mutations/Mb) generally correlates with better ICI efficacy due to a greater chance of generating immunogenic neoantigens (37). High TMB can be observed in some MSS endometrial cancers and smoking-related cervical cancers. However, methods for TMB testing (whole-exome sequencing vs large-panel next-generation sequencing) and cutoff definition have not been fully standardized and remain under investigation. A major barrier to global clinical adoption is the lack of standardization across different sequencing platforms: whole-exome sequencing (WES) and large-panel next-generation sequencing (NGS) yield different TMB values, and no universal cutoff has been validated across cancer types or assays (37).

### 4.4 Immune gene expression signatures

Transcriptional profiling of tumor tissues using technologies such as NanoString or RNA sequencing enables more comprehensive assessment of TIME status (38). Signatures enriched for IFN-γ signaling-related genes such as CXCL9, CXCL10, and IDO1, T-cell inflammatory gene expression profiles (GEP), or genes associated with effector T-cell function have been associated with favorable ICI efficacy across multiple cancer types (38).

### 4.5 Gut microbiome

Accumulating evidence indicates that gut microbiota composition systematically influences host immune status and thereby ICI efficacy (39). Enrichment of specific beneficial bacteria such as Faecalibacterium prausnitzii, Akkermansia muciniphila, and Bifidobacterium species is often associated with better response and longer survival, whereas other taxa such as Bacteroides species may be linked to resistance. Strategies modulating gut microbiota, such as fecal microbiota transplantation and probiotics/prebiotics, are therefore being explored as adjuncts to immunotherapy (39).

### 4.6 Peripheral blood biomarkers

This category includes peripheral T-cell clonality, circulating cytokine levels, and dynamic changes in circulating tumor DNA (ctDNA). Early clearance of ctDNA has been consistently associated with favorable clinical outcomes and represents a promising real-time, non-invasive tool for monitoring treatment response (40).

### 4.7 Emerging biomarker frameworks: beyond conventional markers

#### 4.7.1 T-cell receptor repertoire diversity

High TCR clonality (i.e., expansion of a few dominant T-cell clones) has been associated with favorable ICI responses in endometrial and cervical cancers, whereas low diversity often reflects immune ignorance (41).

#### 4.7.2 Neoantigen quality and clonality

The number of clonal neoantigens (present in all tumor cells) is a better predictor of ICI response than total TMB, as subclonal neoantigens are less likely to be recognized by T cells (42).

#### 4.7.3 Tertiary lymphoid structures

The presence of mature TLS (characterized by CD20^+^ B cell follicles and CD3^+^ T cell zones) in ovarian and cervical cancers correlates with improved ICI outcomes, possibly by facilitating local T cell priming (43).

#### 4.7.4 Spatial immune signatures

Multiplex immunohistochemistry and digital spatial profiling allow simultaneous quantification of multiple immune markers within defined tumor regions (44). A “spatial score” that integrates proximity of CD8^+^ T cells to tumor cells is currently being validated as a predictive biomarker (44).

#### 4.7.5 Peripheral immune profiling

Mass cytometry (CyTOF) and high-dimensional flow cytometry of peripheral blood can capture systemic immune changes, including the emergence of proliferating Ki-67^+^ PD-1^+^ CD8^+^ T cells 3-4 weeks after ICI initiation, which predicts radiographic response (45).

#### 4.7.6 Integrated multi-omics

**Figure 2:**
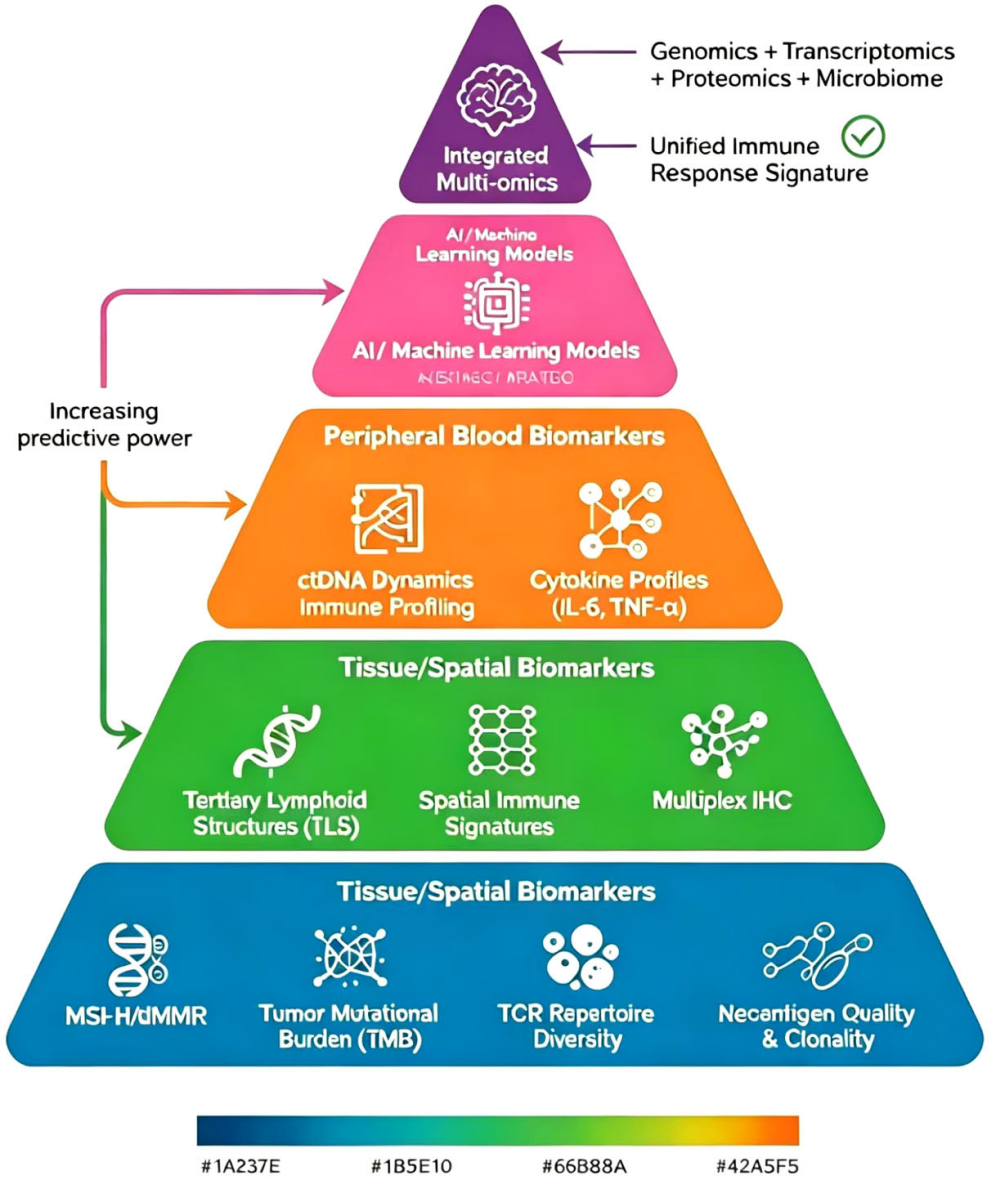
Emerging biomarker frameworks for ICI response prediction in gynecologic cancers. A multi-layer biomarker framework is presented: (1) genomic biomarkers including MSI-H/dMMR, TMB, TCR repertoire diversity, and neoantigen quality/clonality; (2) tissue-based biomarkers including TLS, spatial immune signatures, and multiplex IHC; (3) peripheral blood biomarkers including ctDNA dynamics, peripheral immune profiling, and cytokine profiles; and (4) integrated multi-omics approaches combining genomic, transcriptomic, proteomic, and microbiome data using AI/machine learning models to generate unified predictive signatures.

Machine learning models that combine genomic, transcriptomic, and proteomic data are being developed to produce a unified “immune response signature,” which holds promise for clinical deployment (38) (**Figure 2**).

### 4.8 Single-cell and spatial profiling technologies: unveiling immune heterogeneity

Recent advances in single-cell RNA sequencing (scRNA-seq) and spatial transcriptomics have dramatically improved our understanding of the TIME in gynecologic cancers (44). scRNA-seq studies have uncovered discrete subpopulations of exhausted T cells, pro-tumorigenic macrophages, and cancer-associated fibroblasts that correlate with ICI resistance. Spatial transcriptomics further localizes these populations within the tumor architecture, distinguishing immune-infiltrated “hot” regions from immune-excluded “cold” niches. For instance, a spatial study in ovarian cancer revealed that PD-L1^+^ TAMs physically colocalize with CD8^+^ T cells in certain tumor areas, suggesting a spatially restricted immune suppression mechanism. These technologies are now being integrated into prospective clinical trials to guide patient selection and evaluate dynamic immune changes during treatment (44).

## 5 Current challenges and future perspectives

Despite the promising outlook of combination strategies, their successful translation into clinical practice faces several major obstacles (5, 46).

### 5.1 Complex resistance mechanisms

Resistance to immunotherapy can be primary (intrinsic) or acquired (47). Underlying mechanisms are multifaceted, involving tumor cell-intrinsic factors such as mutations in IFN-γ pathway genes (JAK1/2, STAT), loss of antigen presentation (B2M mutation), dysregulation of PTEN/PI3K pathway, activation of WNT/β-catenin pathway and extrinsic microenvironmental factors such as T-cell exhaustion, upregulation of alternative immune checkpoints (LAG-3, TIM-3), accumulation of suppressor cells, metabolic disturbances such as tryptophan depletion. Overcoming resistance will require development of novel combination regimens targeting these diverse pathways (47). However, a key and often overlooked challenge is the spatial and temporal heterogeneity of these resistance mechanisms. Biopsies from a single site and time point may fail to capture the full spectrum of resistance, leading to incomplete therapeutic targeting. Future trials should mandate longitudinal and multi-regional sampling to delineate the dynamic evolution of resistance (48).

### 5.2 Toxicity management

Combination therapy inevitably increases the incidence, severity, and complexity of immune-related adverse events (irAEs). Common irAEs include cutaneous, gastrointestinal, hepatic, pulmonary, and endocrine toxicities, as well as rare but life-threatening events such as myocarditis. Developing strategies for early detection, accurate diagnosis, and effective management of irAEs while carefully balancing efficacy and safety represents a major challenge for clinicians. Establishing multidisciplinary teams and standardized management protocols is critical. We argue that the current reactive approach to irAEs is insufficient. The field must shift toward developing predictive biomarkers for irAEs analogous to those for treatment efficacy (49). Emerging predictive biomarkers for irAEs: Preliminary studies suggest that elevated baseline levels of pro-inflammatory cytokines such as IL-6 and TNF-α may correlate with a higher risk of grade ≥3 irAEs (50). In addition, pre-existing autoantibodies (e.g., antinuclear antibodies, anti-thyroglobulin antibodies) have been associated with a greater likelihood of immune-related endocrinopathies and dermatitis. While these markers are not yet ready for routine clinical use, they represent promising tools for risk stratification and personalized monitoring (50). Furthermore, the controversial practice of using corticosteroids to manage irAEs requires re-evaluation, as their potent immunosuppressive effects may inadvertently counteract the anti-tumor immunity we aim to foster (49).

### 5.3 Optimization of therapeutic strategies

Key questions remain unanswered regarding optimal selection of combination partners, treatment sequence (concurrent vs sequential), intervention timing (neoadjuvant, adjuvant, maintenance), and treatment duration. For example, is concurrent or sequential chemo-immunotherapy more effective? What is the optimal duration of maintenance immunotherapy? These critical questions require well-designed prospective clinical trials to address. Beyond this, we foresee a paradigm shift: from fixed-duration therapy toward personalized strategies guided by treatment response and minimal residual disease (MRD) monitoring. Using ctDNA to monitor MRD provides a transformative opportunity to personalize treatment duration—escalating therapy at molecular recurrence and de-escalating or discontinuing treatment in sustained molecular remission—thereby maximizing efficacy while minimizing long-term toxicity and cost (51).

### 5.4 Refinement and integration of biomarkers

Single biomarkers are often neither sufficiently sensitive nor specific to enable precise patient selection. The future direction is to integrate multi-omics data (genomics, transcriptomics, proteomics, microbiomics, etc.) using artificial intelligence and machine learning to build comprehensive predictive models for truly personalized therapy (52). However, the feasibility and cost of such comprehensive analyses remain debated; while academically appealing, translation into routine clinical practice faces substantial barriers. The research community must therefore strive to identify the most cost-effective minimal biomarker panels, likely through international collaboration and data sharing (52).

### 5.5 Focus on rare histologic subtypes

Most clinical trials enroll patients with high-grade serous ovarian cancer, endometrioid endometrial adenocarcinoma, or squamous cell carcinoma of the cervix (17). Data on ICI efficacy are extremely scarce for aggressive but rare histologic types such as uterine carcinosarcoma (MMMT), small-cell neuroendocrine carcinoma of the cervix, and gastric-type adenocarcinoma of the cervix, requiring dedicated basket or umbrella trials to fill this gap. We recommend a paradigm shift in future trial design: master protocols should not exclude these rare types but include dedicated cohorts for them. In addition, funding agencies and pharmaceutical companies should increase support for research on these “orphan diseases,” as this is essential to achieving equitable cancer care (17).

### 5.6 Emerging opportunities for rare gynecologic histologies

Aggressive rare subtypes such as uterine carcinosarcoma (MMMT), small-cell neuroendocrine carcinoma of the cervix, and gastric-type cervical adenocarcinoma are often excluded from large phase III trials, leaving their immunobiology poorly understood. However, emerging data suggest potential vulnerabilities. For MMMT, p53 mutations and the epithelial-to-mesenchymal transition (EMT) signature may create a tumor microenvironment permissive to ICI therapy. For small-cell cervical carcinoma, high tumor mutational burden (TMB) and neuroendocrine features may predict sensitivity to PD-1 blockade. Dedicated basket or umbrella trials are urgently needed to prospectively evaluate ICIs in these orphan diseases. **Table 2** summarizes ongoing trials and potential actionable targets for these rare entities (17).

**Table 2:** Characteristics of rare gynecological tumor subtypes and ongoing clinical trials (17).

Rare subtype	Key molecular features	Potential therapeutic targets	Representative ongoing trials (NCT No.)
Uterine Carcinosarcoma (MMMT)	p53 mutations, EMT signature, variable PD-L1 expression	Anti-PD-1/PD-L1, chemoimmunotherapy, anti-angiogenics	NCT03038100 (pembrolizumab); NCT03241745 (atezolizumab)
Small-Cell Neuroendocrine Cervical Carcinoma	High TMB, TP53/RB1 mutations, neuroendocrine marker expression	PD-1 monotherapy, chemo + ICI, PARP inhibitors	NCT04679519 (nivolumab ± ipilimumab)
Gastric-Type Cervical Adenocarcinoma	HER2-negative, p53-mutant, p16-negative, chemo-resistant	Anti-angiogenics, PD-1 inhibitors, anti-Claudin 18.2 antibodies	NCT06061979 (anti-Claudin 18.2 antibody)
Ovarian Clear Cell Carcinoma	ARID1A/PIK3CA mutations, high PD-L1 expression	PI3K/AKT inhibitors, dual PD-1 + CTLA-4 blockade	NCT03514407 (durvalumab + tremelimumab)
Ovarian Mucinous Carcinoma	KRAS mutations, partial HER2 amplification, rare subtype	KRAS G12C inhibitors, anti-HER2, ICIs	NCT06274893 (KRAS G12C inhibitor)

## 6 Discussion

ICI-based combination strategies have fundamentally transformed the treatment landscape of advanced gynecologic malignancies, offering growing numbers of patients the prospect of long-term survival (6, 18, 21–27, 30). Combinations of ICIs with conventional chemotherapy, anti-angiogenic agents, PARP inhibitors, radiotherapy, and other targeted therapies act through synergistic modulation of the tumor immune microenvironment to achieve greater-than-additive anti-tumor potential (4, 6). Recent phase III trials including KEYNOTE-775, KEYNOTE-B96, KEYNOTE-826, KEYNOTE-A18, BEATcc, and DUO-O have provided pivotal evidence for these regimens (18, 21–25, 30); negative results from trials such as LEAP-001 and JAVELIN Ovarian 100 remind us that challenges persist and biomarker-guided patient selection is essential (23, 26).

Accordingly, the central question in the field is no longer simply whether these combinations work, but how to optimize them in clinical practice. We now face the more refined challenge of selecting the most effective regimen for the right patient and determining the optimal timing of administration (6). Future breakthroughs will undoubtedly depend on deeper understanding of the complexity of the tumor immune cycle and microenvironment, development and validation of a reliable multi-dimensional biomarker system, and continuous refinement of therapeutic strategies through well-designed and rigorously conducted clinical trials. This collective effort will ultimately guide gynecologic oncology into a new era of precise and personalized immunotherapy (6).

### 6.1 Cellular components of the tumor immune microenvironment across gynecologic cancers

**Figure 3:**
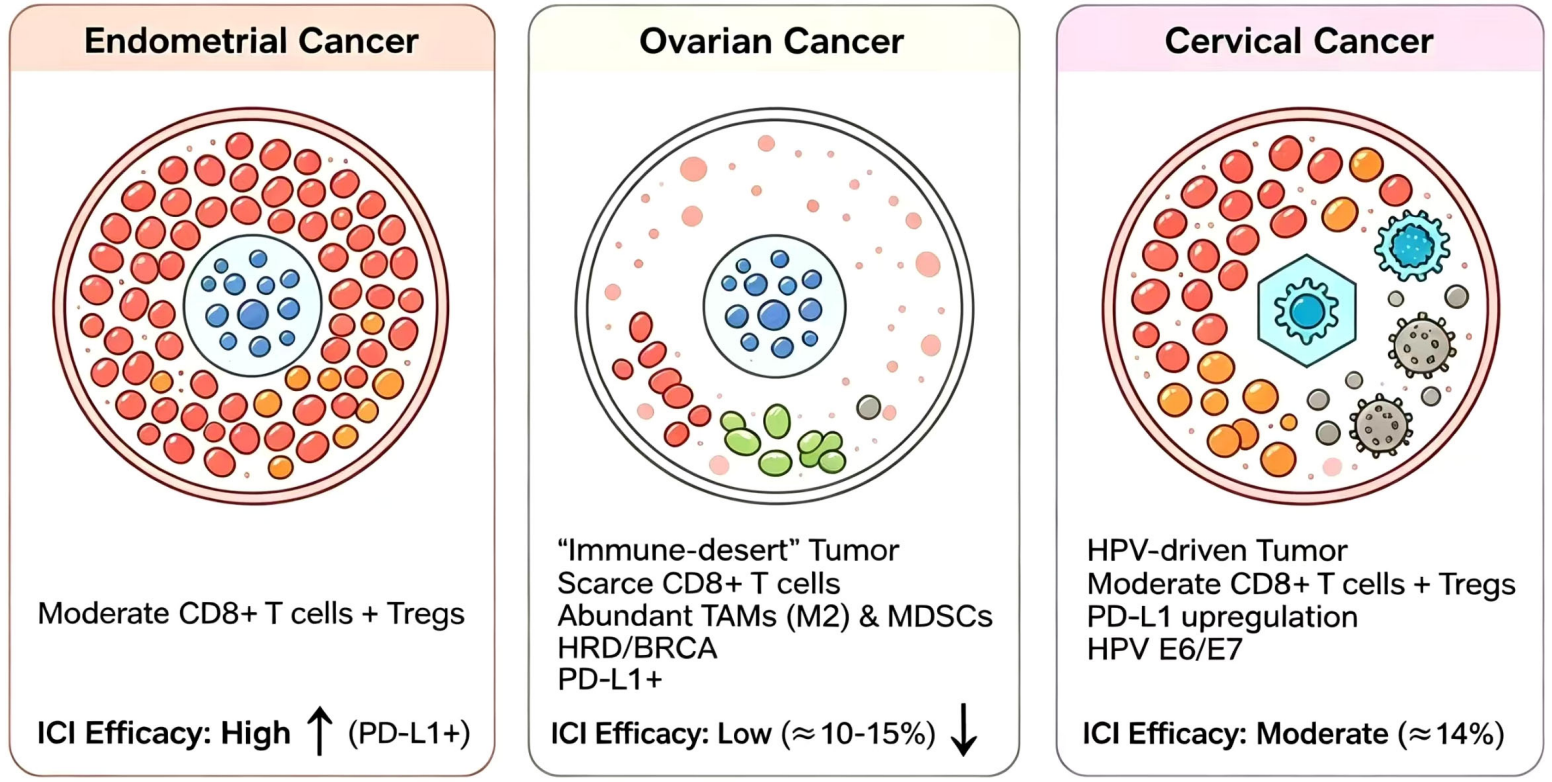
Distinct immune microenvironment landscapes of major gynecologic cancers. Endometrial cancer (left) exhibits MSI-H/dMMR-driven “hot” tumors with abundant CD8^+^ T cells and Tregs; ovarian cancer (middle) is characterized by an “immune-desert” phenotype with scarce CD8^+^ T cells and abundant TAMs (M2) and MDSCs; cervical cancer (right) is HPV-driven with moderate CD8^+^ T-cell infiltration and PD-L1 upregulation. ICI efficacy varies from high (endometrial MSI-H/dMMR) through moderate (cervical) to low (ovarian).

The TIME is shaped not only by checkpoint molecules but also by a complex network of immunosuppressive cell populations (5, 13) (**Figure 3**). Tumor-associated macrophages (TAMs), particularly the M2 phenotype, are abundant in ovarian cancer and secrete IL-10 and TGF-β, promoting immune exclusion and drug resistance (13, 53). Myeloid-derived suppressor cells (MDSCs) accumulate in all three major gynecologic cancers and suppress T cell function via arginase and reactive oxygen species (54). Cancer-associated fibroblasts (CAFs) create a physical barrier to T cell infiltration by depositing dense extracellular matrix and secreting CXCL12 (55). Regulatory T cells (Tregs) are enriched in cervical and ovarian cancers, where they dampen effector T cell responses (13, 54). Exhausted CD8+ T cells (PD-1^+^, TIM-3^+^, LAG-3^+^) are a hallmark of chronic antigen stimulation and correlate with poor ICI response, particularly in platinum-resistant ovarian cancer (56).

Understanding the relative abundance and functional states of these populations across tumor types will be crucial for designing rational combination strategies (13, 53–56).
